# Combining directed evolution of pathway enzymes and dynamic pathway regulation using a quorum-sensing circuit to improve the production of 4-hydroxyphenylacetic acid in *Escherichia coli*

**DOI:** 10.1186/s13068-019-1438-3

**Published:** 2019-04-23

**Authors:** Yu-Ping Shen, Lai San Fong, Zhi-Bo Yan, Jian-Zhong Liu

**Affiliations:** Institute of Synthetic Biology, Biomedical Center, Guangdong Province Key Laboratory of Improved Variety Reproduction in Aquatic Economic Animals, School of Life Sciences, Sun Yat-sen University, Guangzhou, 510275 China

**Keywords:** 4-Hydroxyphenylacetic acid, Directed evolution, Dynamic pathway regulation, Quorum-sensing system, *Escherichia coli*

## Abstract

**Background:**

4-Hydroxyphenylacetic acid (4HPAA) is an important building block for synthesizing drugs, agrochemicals, biochemicals, etc. 4HPAA is currently produced exclusively via petrochemical processes and the process is environmentally unfriendly and unsustainable. Microbial cell factory would be an attractive approach for 4HPAA production.

**Results:**

In the present study, we established a microbial biosynthetic system for the de novo production of 4HPAA from glucose in *Escherichia coli*. First, we compared different biosynthetic pathways for the production of 4HPAA. The yeast Ehrlich pathway produced the highest level of 4HPAA among these pathways that were evaluated. To increase the pathway efficiency, the yeast Ehrlich pathway enzymes were directedly evolved via error-prone PCR. Two phenylpyruvate decarboxylase ARO10 and phenylacetaldehyde dehydrogenase FeaB variants that outperformed the wild-type enzymes were obtained. These mutations increased the in vitro and in vivo catalytic efficiency for converting 4-hydroxyphenylpyruvate to 4HPAA. A tunable intergenic region (TIGR) sequence was inserted into the two evolved genes to balance their expression. Regulation of TIGR for the evolved pathway enzymes further improved the production of 4HPAA, resulting in a 1.13-fold increase in titer compared with the fusion wild-type pathway. To prevent the toxicity of a heterologous pathway to the cell, an Esa quorum-sensing (QS) circuit with both activating and repressing functions was developed for inducer-free productions of metabolites. The Esa-P_esaR_ activation QS system was used to dynamically control the biosynthetic pathway of 4HPAA in *E. coli*, which achieved 17.39 ± 0.26 g/L with a molar yield of 23.2% without addition of external inducers, resulting in a 46.4% improvement of the titer compared to the statically controlled pathway.

**Conclusion:**

We have constructed an *E. coli* for 4HPAA production with the highest titer to date. This study also demonstrates that the combination of directed evolution of pathway enzymes and dynamic pathway regulation using a QS circuit is a powerful strategy of metabolic engineering for the productions of metabolites.

**Electronic supplementary material:**

The online version of this article (10.1186/s13068-019-1438-3) contains supplementary material, which is available to authorized users.

## Background

Phenolic acids are aromatic acids that contain a phenol ring and at least one organic carboxylic acid group. Phenolic acids play an important role in human health and have wide applications in food, cosmetic and pharmaceutical industries. 4-Hydroxyphenylacetic acid (4HPAA) has received much attention because of its numerous applications. 4HPAA is used in the synthesis of penicillin G, atenolol, benzoprofen, and agrochemicals, etc. [1, 2]. 4HPAA is an active component of *Rhodiola rosea* [2] and the Chinese herbs *Aster tataricus* (fan hun cao). *Aster tataricus* is widely used in China for the treatment of pneumonia, HBV, and carcinomas [3–5]. Furthermore, 4HPAA possesses anxiolytic [6], antiplatelet [7] and hepatoprotective [8] properties. In addition, 4HPAA was considered as a potential hypopigmenting agent [9] and an inhibitor of hypertonicity and hypoxia [10].

4HPAA can be obtained by chemical synthesis from different substrates such as anisol, *p*-cresol, phenol, benzyl phenyl ether, or hydroxymandelic acid [11, 12]. However, the chemical routes have some drawbacks, including a requirement of elevated temperatures and pressures for the reaction, and the use of expensive solvents. Thus, biotechnological approach has been explored as an alternative. A biotransformation reaction using nitrilase has been used for 4HPAA production [2]. Koma et al. [1] engineered an *Escherichia coli* for the production of 4HPAA from glucose. Overexpression of the indole-3-pyruvate/phenylpyruvate decarboxylase gene *ipdC* from *Azospirillum brasilense* NBRC102289, and the phenylacetaldehyde dehydrogenase gene *feaB* from *E. coli* in a tyrosine overproducing *E. coli* strain resulted in the production of 6.1 mM (0.93 g/L) 4HPAA with a yield of 13.2% (mol/mol) in a shake flask culture. However, the titer of 4HPAA in engineered microorganisms is much lower than that of other aromatic compounds [13, 14]. Thus, further work is required to increase the production of 4HPAA.

Quorum-sensing (QS) systems has been considered as an auto-induction system that is regulated by cell density. The Lux and Esa QS systems are the main QS systems that have been reported to date. Recently, an Esa QS system from *Pantoea stewartii* has been engineered to automatically downregulate the competing pathway, significantly improving the production of myo-inositol, glucaric acid and shikimic acid [15].

**Fig. 1:**
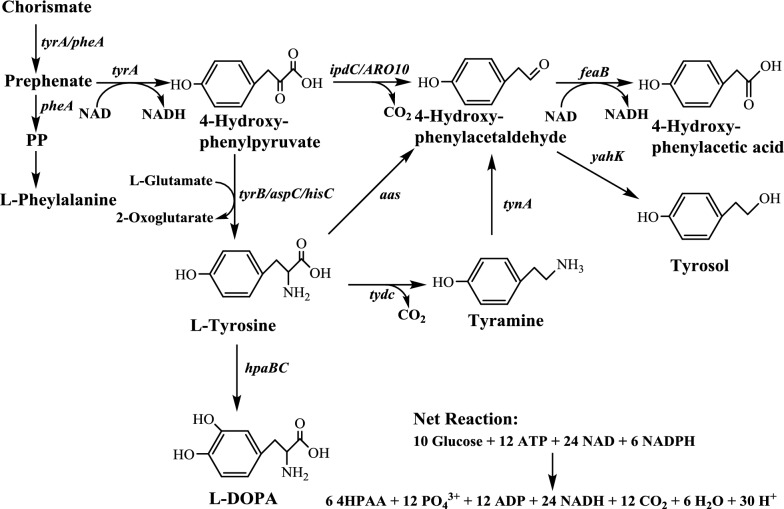
Biosynthetic pathway of 4-hydroxyphenylacetic acid (4HPAA). *tyrA/pheA* fused chorismate mutase/prephenate dehydrogenase gene, *ipdC* indole-3-pyruvate/phenylpyruvate decarboxylase gene from *Azospirillum brasilense*, *ARO10* phenylpyruvate decarboxylase gene from *Saccharomyces cerevisiae*, *tyrB* tyrosine aminotransferase gene, *aspC* aspartate aminotransferase gene, *hisC* histidinol-phosphate aminotransferase gene, *hpaBC* 4-hydroxyphenylacetate 3-monooxygenase gene from *E. coli* W, *aas* aromatic aldehyde synthase gene from *Petroselinum crispum*, *tydc* tyrosine/DOPA decarboxylase gene from *Pseudomonas putida* KT2440, *tynA* tyramine oxidase gene, *feaB* phenylacetaldehyde dehydrogenase gene, *yahK* NADPH-dependent aldehyde reductase gene

In this study, we first compared different biosynthetic pathways of 4HPAA (Fig. 1). Then, the biosynthetic pathway enzymes of 4HPAA were directedly evolved and mediated using a tunable intergenic region (TIGR) sequence. Finally, the TIGR-mediated biosynthetic pathway was dynamically regulated using a quorum-sensing circuit. The resulting *E. coli* produced 17.39 ± 0.26 g/L 4HPAA without addition of external inducers, which is the highest value reported to date.

## Methods

### Strains, plasmids and primers

The bacterial strains, plasmids, and primers used in this study are listed in Table 1. *E. coli* DH5α was used for gene cloning in this study. The L-DOPA overproducing strain *E. coli* DOPA-30N [16] was used as the host strain for the production of 4HPAA.

**Table 1:** Strains, plasmids and primers used in this study

Name	Description	Source/purpose
Strain		
*E. coli* DH5α	supE44 Δ(lacZYA-argF) U169 (Φ80lacZ ΔM15) hsdR17 recA endA1 gyrA96 thi-1 relA1	Invitrogen
*E. coli* BW 25113	*lacI* ^q^ *rrnB*_T14_ *ΔlacZ*_WJ16_ *hsdR514 ΔaraBAD*_AH33_ *ΔrhaBAD*_LD78_	[17]
*E. coli* DOPA-30N	L-DOPA overproducer	[16]
Plasmid		
pBbB2k-GFP	Expressing plasmid, BglBrick vectors, tet promoter, pBBR1 *ori*, kan^r^, Addgene plasmid #35345	[18]
pBbB2k-ipdC-L-feaB	pBbB2k derivatives harboring the fusion gene cluster of *ipdC* from *Azospirillum brasilense* and *feaB* from *E. coli* with a (GSG)_2_ linker	This study
pBbB2k-ARO10-L-feaB	pBbB2k derivatives harboring the fusion gene cluster of *ARO10* from *Saccharomyces cerevisiae* and *feaB* from *E. coli* with a (GSG)_2_ linker	This study
pBbB2k-aas-L-feaB	pBbB2k derivatives harboring the fusion gene cluster of *aas* from *Petroselinum crispum* and *feaB* from *E. coli* with a (GSG)_2_ linker	This study
pBbB2k-tydc-L-tynA-L-feaB	pBbB2k derivatives harboring the fusion gene cluster of *tydc* from *Pseudomonas putida* KT2440, *tynA* and *feaB* from *E. coli* with a (GSG)_2_ linker	This study
pBbB2k-ARO10*- L-feaB*	pBbB2k derivatives harboring the fusion gene cluster of the evolved *ARO10*_*6D5*_ from *S. cerevisiae* and *feaB*_*2E1*_ from *E. coli* with a (GSG)_2_ linker	This study
pBbB2k-ARO10*-feaB*	pBbB2k derivatives harboring the gene cluster of the evolved *ARO10*_*6D5*_ from *S. cerevisiae* and *feaB*_*2E1*_ from *E. coli*, one operon	This study
pBbB2k-ARO10*-TIGR-feaB*	pBbB2k derivatives harboring the TIGR-mediated gene cluster of the evolved *ARO10*_*6D5*_ from *S. cerevisiae* and *feaB*_*2E1*_ from *E. coli*	This study
pZBK	BglBrick/ePathBrick expression vector, pBBR1 *ori*, P37 promoter, kan^r^	[19]
pZBK-P_esaS_IC-P_esaR_AS	Quorum-sensing plasmid, pBBR1 *ori*, P37 promoter, kan^r^	This study
pZBK-P_esaR_AS-4HPAA	pZBK-P_esaS_IC-P_esaR_AS harboring the TIGR-mediated gene cluster of the evolved *ARO10*_*6D5*_ from *S. cerevisiae* and *feaB*_*2E1*_ from *E. coli*	This study
Primer		
FeaBF	CCGGAATTCAAAAGATCTGGTATGACAGAGCCGCATGTAGCAG (*Eco*RI, *Bgl*II)	PCR for *feaB*
FeaBR	CGCGGATCCTTAATACCGTACACACACCGACTTAGTTTCACACCAAC (*Bam*HI)	
TynAF	CCGGAATTCAAAAGATCTGGTATGGGAAGCCCCTCTCTGTATTCTG (*Eco*RI, *Bgl*II)	PCR for *tynA*
TynAR	CGCGGATCCACCTGAACCCTTATCTTTCTTCAGCGCCC (*BamH*I)	
ARO10F	CCGGAATTCAAAAGATCTTTTCGGAATTAAGGAGGTAATAAATATGGCACCTGTTACAATTG (*Eco*RI, *Bgl*II)	PCR for *ARO10*
ARO10R	CGCGGATCCACCACTACCTTTTTTATTTCTTTTAAGTG (*Bam*HI)	
AROF	GATAGAGAAAAGAATTCAAAAGATCTTTTCGGAATTAAGGAGG (*Eco*RI, *Bgl*II)	error-prone PCR for*ARO10*-*feaB*
FeaR	TACTCGAGTTTGGATCCTTAATACCGTACACACACCGACTT (*Bam*HI)	
epAROF	ACCTTCGATTCCGACCTCATTAAGC	error-prone PCR for *ARO10*
epAROR	CGCGGATCCTTAATACCGAACACACACCGACTTAGTTTCACACCAAC	
TIGRs-F(X)	CTAGCTAGCGCCTAGCAAGATCTCCTGATCCCGGTGC (*Nhe*I)	PCR for TIGRs
TIGRs-R(A)	TTTTCCTTTTGCGGCCGCGGATACAGTATCTGCGGTACCCTAG (*Not*I)	
Qaro10F	CGACACCTTGTTTCCAACCG	qRT-PCR for ARO10
Qaro10R	TGAAGTCACCAGGAACACCG	
QfeaBF	GCCAACGCTGGTGGTAAATC	qRT-PCR for feaB
QfeaBR	GCCTCTTCTCCATCCGCTAC	

### Construction of plasmids

The codon-optimized *ipdC* (GenBank accession number X99587) from *A. brasilense* NBRC102289, *tydc* (encoding tyrosine/DOPA decarboxylase, GenBank accession number AE015451) from *Pseudomonas putida* KT2440, and *aas* (encoding aromatic aldehyde synthase, GenBank accession number M96070) from *Petroselinum crispum* (encoding aromatic aldehyde synthase) were synthesized by GENEWIZ, Inc. (Suzhou, China). The *feaB* (encoding phenylacetaldehyde dehydrogenase) and *tynA* (encoding tyramine oxidase) genes were amplified from *E. coli*. The *ARO10* (encoding phenylpyruvate decarboxylase) was amplified from *Saccharomyces cerevisiae*. The protein fusion is a common approach of substrate channeling for coordinating expression of cascade enzymes [19–21]. Thus, a linker encoding (GSG)_2_ was inserted into the two genes, and the TAA stop codon of the front gene was deleted. The fusion pathway was cloned into pBbB2k-GFP to obtain pBbB2k-ipdC-L-feaB, pBbB2k-ARO10-L-feaB, pBbB2k-tydc-L-tynA-L-feaB, pBbB2k-aas-L-feaB, respectively.

### Generation of random mutagenesis libraries using error-prone PCR and screening

MEGAWHOP-PCR [22] was performed for the construction of libraries. The random mutagenesis libraries of the fusion ARO10-feaB gene cluster were constructed through error-prone PCR. Primer pairs AROF/FeaR were used for amplification the ARO10-feaB gene cluster using pBbB2k-ARO10-L-feaB as a template. The PCR reaction mixture (50 μL) consisted of 5 mM MgCl_2_, 0.3 mM MnCl_2_, 0.2 mM each of dATP, dGTP, dCTP and dTTP, and 2.5 U of rTaq DNA polymerase. The PCR products were then used as megaprimers to perform MEGAWHOP-PCR using pBbB2k-ARO10-L-feaB as a template. Following the MEGAWHOP-PCR, *Dpn*I digestion (20 U) of the surplus template in the PCR reaction mixture was performed at 37 °C overnight, then *Dpn*I was inactivated at 80 °C for 20 min. The PCR products were transformed into *E. coli* DH5a cells, plated on LB agar, and then pooled together after overnight growth to create a liquid library.

The mutant plasmid was recovered from the library and transferred into the L-DOPA overproducer *E. coli* DOPA-30N. Then, the resulting transformants were plated on LB agar with 50 mg/mL kanamycin and 120 ng/mL anhydrotetracycline. After overnight growth, individual colonies were inoculated into a 48-well deep-well microplate (4.6 mL) containing 1 mL of the fermentation medium and then incubated at 37 °C and 1000 rpm for 72 h on a MBR-420FL shaker (TAITEC Corporation, Saitama-ken, Japan). Because L-DOPA can be easily oxidized to dopachrome and then polymerized non-enzymatically to form melanin, the culture broth of the host strain *E. coli* DOPA-30N became black [16]. Because 4HPAA shares the same precursor (tyrosine) with L-DOPA, light color cultures were selected for further shake flask analysis of 4HPAA production.

### Creating TIGR libraries and screening

To replace the linker between ARO10* and feaB* in pBbB2k-ARO10*-L-feaB* with a two-restriction enzyme sequence, the ARO10* and feaB* genes were amplified from pBbB2k-ARO10*-L-feaB*. The two genes were then cloned into pBbB2k-GFP to obtain pBbB2k-ARO10*-feaB*.

TIGRs were synthesized using PCR to assemble the oligonucleotides into chimeric DNA sequences as described by Pfleger et al. [23] and Li et al. [19]. The assembled products were purified using a nucleotide removal column, amplified using the end-specific primers TIGRs-F(X)/TIGRs-R(A), and then cloned into the *Nhe*I/*Not*I sites of pBbB2k-ARO10*-feaB* to obtain the plasmid libraries pBbB2k-ARO10*-TIGRs-feaB*. The plasmid libraries were transferred into component DOPA-30N to generate the mutant library.

The TIGR library was plated on LB agar plates containing 50 μg/mL kanamycin and 120 ng/mL anhydrotetracycline. The plates were then incubated at 30 °C overnight. Single colonies were inoculated into a 48-well deep-well microplate (4.6 mL) containing 1 mL of fermentation medium, and then incubated at 30 °C and 1000 rpm for 72 h on a MBR-420FL shaker (TAITEC, Japan). Lighter color cultures were selected for further shake flask analysis of 4HPAA production.

### Construction of a quorum-sensing circuit

The quorum-sensing fragment containing the signal molecule acyl-homoserine lactone (AHL) synthase gene *esaI*, the transcriptional regulator *esaR*^*I70V*^, and P_easS_ and P_esaR_ promoters was synthesized by GENEWIZ, Inc (Suzhou, China) and then cloned into the *Mlu*I/*Sal*I of pZBK [19] to obtain the plasmid pZBK-P_easS_IC-P_easR_AS (Additional file 1: Figure S1A). The TIGR-mediated gene cluster of the evolved *ARO10* and *feaB* was cloned into the multiple cloning site 2 (MCS2) of pZBK-P_easS_IC-P_easR_AS to obtain pZBK-P_esaR_AS-4HPAA.

### Production of 4HPAA

A single colony was inoculated into 5 mL of LB medium in a falcon tube then cultured overnight at 30 °C. The overnight seed culture was then inoculated into 50 mL of fermentation medium with a starting OD_600_ of 0.1. The fermentation medium contained (per liter) tryptone (10 g), yeast extract (5 g), NaCl (10 g), glucose (40 g), KH_2_PO_4_ (0.6 g), Na_2_HPO_4_·7H_2_O (2.56 g), and 10 mL of trace element solution. The trace element solution contained (per liter) FeSO_4_·7H_2_O (10 g), ZnSO_4_·7H_2_O (2.2 g), MnSO_4_·4H_2_O (0.58 g) CuSO_4_·5H_2_O (1 g), (NH_4_)_6_Mo_7_O_24_·4H_2_O (0.1 g), and Na_2_B_4_O_7_·10H_2_O (0.2 g). The pH of the medium was adjusted to 7.0. The main cultures were incubated at 30 °C and 200 rpm. When the OD_600_ reached 2.5, the cultures were induced with 120 ng/mL anhydrotetracycline. After induction, the cultures were incubated at 30 °C and 200 rpm for 72 h.

Fed-batch fermentation was carried out in a 2 L bioreactor (MiniBox 2L*2 Parallel Bioreactor System, T&J Bio-engineering (Shanghai) Co. LTD, Shanghai, China) containing 1.2 L of fermentation medium with an initial OD_600_ of approximately 0.1. Fermentation was carried out at 30 °C with an airflow of 1.2 L/min and a starting agitation rate of 400 rpm. For *E. coli* containing the statically controlled pathway, 120 ng/mL anhydrotetracycline was added as an inducer after 6 h. The pH was controlled at 7.0 by automatic addition of NH_4_OH. Dissolved oxygen was maintained above 25% by adjusting the agitation rate. The feed solution (pH 7.0,) contained 500 g/L glucose and 30 g/L MgSO_4_·7H_2_O. The feed was introduced continuously into the fermenter using a pH-stat feeding strategy. Once the glucose was exhausted, the pH rose rapidly. When the pH was greater than 7.0 by 0.1 of a pH unit, the feed was automatically added to the fermenter. Samples were periodically withdrawn, and the following parameters were measured: OD_600_, residual glucose concentrations, and 4HPAA concentrations. Fermentation experiments were performed in duplicate.

### Catalytic efficiency assays

For in vitro activity assay, *E. coli* DOPA-30N harboring pBbB2k-ARO10-L-feaB or pBbB2k-ARO10*-L-feaB* were pre-inoculated in 5 mL of LB medium overnight and then inoculated into 50 mL the fermentation medium containing kanamycin. The cultures were incubated at 30 °C and 200 rpm until OD_600_ reached 2.5 and then induced with 120 ng/mL anhydrotetracycline. After 44 h, the culture broth was disrupted directly using an Ultra-high Pressure Cell Disrupter (JN-3000Plus, Guangzhou Juneng Nano & Bio Technology Co. LTD, China), and then centrifuged to remove cell debris. The supernatant was selected as a crude enzyme solution for catalytic efficiency assay. The total protein concentration in the crude extract was determined by a Bradford assay using a Bio-Rad protein assay kit (Bio-Rad Laboratories, Hercules, CA, USA). The catalytic efficiency assay was conducted as follows: 4-hydroxyphenylpyruvic acid of 10 mM was added to the crude enzyme. The reaction was conducted at 37 °C for 6 h. Then, the reaction mixture was immediately placed on ice broth to stop the reaction. The product (4HPAA) concentration in the reaction solution was determined immediately by HPLC.

For in vivo activity assay, *E. coli* BW25113 was transformed with pBbB2k-ARO10-L-feaB and pBbB2k-ARO10*-L-feaB*, respectively. Single colonies were pre-inoculated into 5 mL of LB medium containing kanamycin and cultured overnight at 30 °C and 200 rpm. A 500 μL overnight cultures were inoculated into 50 mL the fermentation medium containing kanamycin. The cultures were incubated at 30 °C and 200 rpm until OD_600_ reached 2.5 and then induced with 120 ng/mL anhydrotetracycline at 37 °C and 200 rpm. Three hours after induction, 4-hydroxyphenylpyruvic acid was added to a final concentration of 1.0 g/L and incubated at 30 °C and 200 rpm for 6 h. Growth and the concentration of 4HPAA were analyzed.

### Assay

Growth was monitored by measuring the optical density at 600 nm. 4HPAA in the supernatants was analyzed using a Shimadzu HPLC system (LC-20A, Shimadzu, Japan) equipped with an Inertsil ODS-SP column (5 μm, 4.6 × 150 mm, GL Sciences Inc., Tokyo, Japan). The mobile phase was 0.2% TFA in methanol, and the flow rate was 0.5 mL/min at 30 °C. The methanol concentration was increased from 14 to 45% for 20 min, and then decreased to 14%. This concentration was then maintained for 10 min. A photodiode array detector (SPD-M20A) operating at 222 nm was used, and a standard curve was constructed from serial dilutions of a standard stock solution. Glucose concentrations were determined using glucose oxidase and a glucose assay kit (Shanghai Rongsheng Biotech Corporation, Shanghai, China).

### Statistical analysis

All experiments were conducted in triplicate, and the data were averaged and presented as the mean ± standard deviation. One-way analysis of variance followed by Tukey’s test was used to determine significant differences using the OriginPro (version 7.5) package. Statistical significance was defined as *p *< 0.05.

## Results and discussion

### Screening biosynthetic pathways

Three biosynthetic pathways (Fig. 1) have been developed for the production of tyrosol; namely, the Ehrlich pathway via the *ipdC* [1] and the yeast *ARO10* [24, 25], the *aas* (encoding aromatic aldehyde synthase) pathway [26] and the *tydc*-*tyo* (encoding tyrosine decarboxylase and tyramine oxidase, respectively) pathway [27]. Because 4HPAA shares the same precursor, 4-hydroxyphenylacetaldehyde (4HPAAL), with tyrosol, we first constructed these pathways and evaluated their 4HPAA productions (Fig. 1). These pathways were then introduced into the L-DOPA overproducing *E. coli* strain DOPA-30N for shake flask analysis of 4HPAA. No L-DOPA was detected in the four engineered strains, indicating that, the rate of 4HPAA production was higher than that for L-DOPA (data not shown). Thus, *E. coli* DOPA-30N was used as the parent strain for the production of 4HPAA in this study. The introduction of all pathways resulted in the production of 4HPAA (Table 2), indicating that these pathways can be used for 4HPAA production. Moreover, the yeast Ehrlich pathway produced the highest titer of 4HPAA (Table 2). The recombinant strain harboring the yeast Ehrlich pathway produced 3.08 ± 0.05 g/L 4HPAA with a molar yield of 13.4%. The same result was obtained for tyrosol. The yeast Ehrlich pathway also exhibited the highest biosynthetic efficiency for tyrosol production among the four pathways [1, 24–27]. Therefore, the yeast Ehrlich pathway was selected as the biosynthetic pathway for the production of 4HPAA in this study.

**Table 2:** 4-Hydroxyphenylacetic acid production in *E. coli* DOPA-30N harboring different biosynthetic pathways

Host strain	Plasmid	OD600	Titer (g/L)	Yield (%, mol/mol)
*E. coli* DOPA-30N	pBbB2k-ipdC-L-feaB	16.28 ± 0.45	2.32 ± 0.05	11.8 ± 0.2
	pBbB2k-ARO10-L-feaB	17.36 ± 0.19	3.08 ± 0.05	13.4 ± 0.2
	pBbB2k-aas-L-feaB	17.13 ± 1.44	2.11 ± 0.06	9.3 ± 0.2
	pBbB2k-tydC-L-tynA-L-feaB	15.44 ± 0.42	2.41 ± 0.04	12.1 ± 0.2
	pBbB2k-ARO10*-L-feaB*	16.68 ± 0.35	5.64 ± 0.06	22.9 ± 0.3
	pBbB2k-ARO10*-TIGR-feaB*	17.12 ± 0.32	6.58 ± 0.16	27.5 ± 0.3

Data represent the means of three replicates and standard deviations

Although only *ipdC* Ehrlich pathway was reported for the production of 4HPAA in literatures, our results demonstrated that three other pathways based on the reported tyrosol biosynthetic pathways can be used for the production of 4HPAA. Moreover, the 4HPAA titer that was obtained via our yeast Ehrlich pathway was 1.33-fold as high as that obtained via the *ipdC* Ehrlich pathway.

### Directed evolution of the biosynthetic pathway

**Fig. 2:**
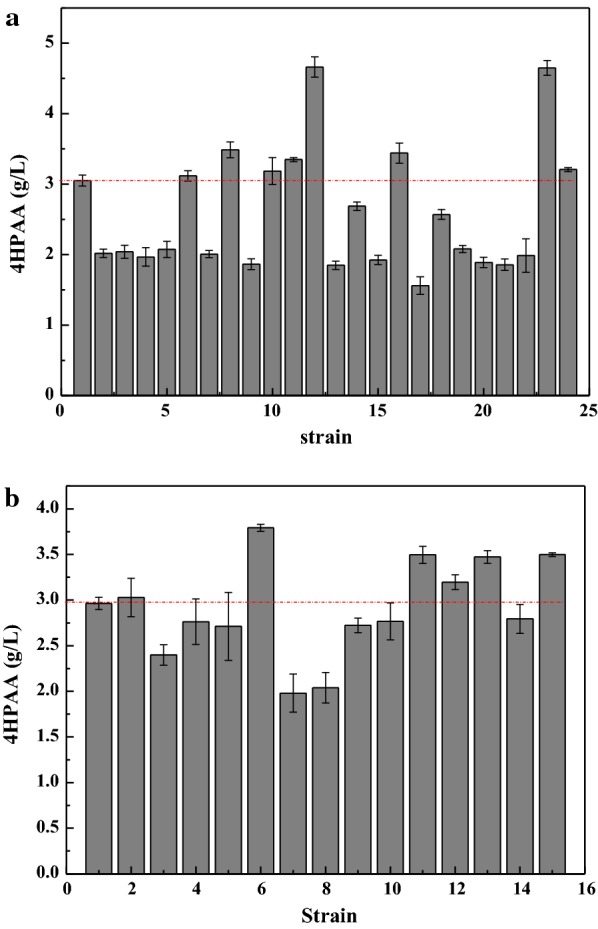
4HPAA production by *E. coli* DOPA-30N harboring mutant gene clusters from error-prone PCR. **a** The mutant gene clusters caused by the co-evolution of the *ARO10*-L-*feaB*. **b** The mutant gene clusters caused by the single-evolution of *ARO10*. Cells were grown at 30 °C and 200 rpm for 72 h. See “Methods” for other details. *E. coli* DOPA-30N (pBbB2k-ARO10-L-feaB) (strain no. 1) was set the control. The data represent the means of three replicates, and error bars represent standard deviations

Independent of the enzyme structure and catalytic mechanism, directed evolution of enzyme is widely used to alter the coding sequence and improve the catalytic activities of enzymes. Thus, to increase the efficiency of the biosynthetic pathway, the fusion gene cluster of *ARO10*-*feaB* was directedly co-evolved by error-prone PCR. L-DOPA can be easily oxidized to dopachrome and then polymerized non-enzymatically to form the black pigment melanin [28]. Because the preparation of 4HPAA utilizes the same precursors (4-hydroxyphenylpyruvate/tyrosine) as L-DOPA (Fig. 1), 4HPAA biosynthesis competes with L-DOPA. The higher the activity of the fusion gene cluster, the lower the production of L-DOPA, hence, the culture broth becomes lighter. A total of 792 colonies were used for the assay of 4HPAA production in deep-well microplate cultures. Twenty-three colonies with lighter colors were selected for further shake flask analysis. As shown in Fig. 2a, strains no. 12 and 23 produced the highest level of 4HPP (4.66 ± 0.14 and 4.65 ± 0.10 g/L, respectively). These mutant plasmids, denoted as 2E1 and 4F3, were isolated and sent for sequencing. The sequencing results are presented in Additional file 1: Table S1. In plasmid 2E1, one base mutant (C1869T) in *ARO10* and three base mutants (T72G, T1195C, and A1491T) were observed in *feaB*. In plasmid 4F3, two base mutants (A1682G and A1842G) in *ARO10* and three base mutants (C165G, A609T, and T1296) were observed in *feaB*. None of the base mutants in *ARO10* produced an amino acid change. One amino acid mutant of *feaB* was observed in plasmid 2E1 (I24M) and 4F3 (N55K).

Because no amino acid mutant in *ARO10* was observed after the co-evolution of the *ARO10*-*feaB*, the *ARO10* in pBbB2k-ARO10-L-feaB was single-evolved using error-prone PCR. Of 900 colonies used for the assay in deep-well microplate cultures, twelve colonies with lighter colors were selected for further shake flask analysis. As shown in Fig. 2b, strains no. 6, 11, 13, and 15 produced higher levels of 4HPAA than the wild-type strain. The mutant plasmids, denoted as 6D5, 9F5, 10A3, and 10G5, were isolated and sent for sequencing. DNA sequencing revealed some base mutations and amino acid mutations in *ARO10* (Additional file 1: Table S1). Two amino acid mutations (F138L and D218G) were observed in plasmid 6D5, and one mutation (V451I) was observed in plasmid 10A3.

**Fig. 3:**
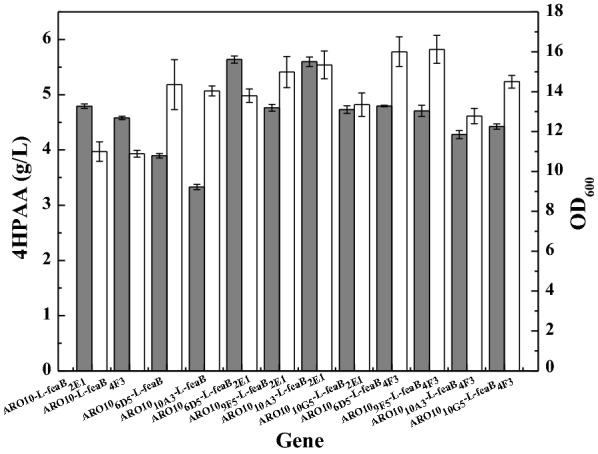
4HPAA production by *E. coli* DOPA-30N harboring the fusion gene cluster of different combinations of the evolved *S. cerevisiae ARO10* and *E. coli feaB*. Growth (white bar); 4HPAA concentration (gray bar). Cells were grown at 30 °C and 200 rpm for 72 h. See “Methods” for other details. The data represent the means of three replicates, and error bars represent standard deviations

To investigate the effects of these mutated genes on the production of 4HPAA, we combined these mutated genes to create a biosynthetic pathway and introduced the pathway into *E. coli* DOPA-30N. Figure 3 shows that mutations of two genes indeed increased the production of 4HPAA. The engineered strain harboring both mutated genes produced higher levels of 4HPAA than the strain that contained only one mutated gene. The combination of the mutated *ARO10* from plasmid 6D5 with the mutated *feaB* from plasmid 2E1 resulted in the highest titer of 4HPAA (5.64 ± 0.06 g/L). This combination resulted in approximately a 90% increase in the production of 4HPAA compared to the wild-type genes.

**Fig. 4:**
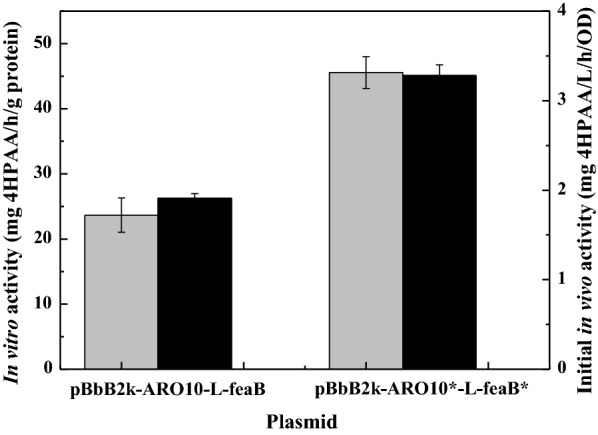
In vitro (gray bar) and initial in vivo (black bar) catalytic efficiency of the pathway. The data represent the means of three replicates, and error bars represent standard deviations

To evaluate the applicability of the evolved pathway enzymes in microbial production of 4HPAA, we first performed in vitro crude extract enzyme assays to measure their catalytic activities. 4-Hydroxyphenylpyruvic acid of 10 mM was added into the cell crude extracts of *E. coli* DOPA-30N harboring the wild-type or mutant plasmid, respectively. The catalytic efficiencies were measured by testing the 4HPAA formation rate. As shown in Fig. 4, directed evolution resulted in an increase in the in vitro catalytic efficiency of the pathway to 45.55 ± 2.43 mg/h/g protein from 23.67 ± 2.66 mg/h/g protein (Fig. 4), indicating that the evolved pathway was more efficient at converting 4-hydroxyphenylpyruvate to 4HPAA than the wild-type pathway in vitro.

Then, the initial in vivo efficiency of converting 4-hydroxyphenylpyruvic acid to 4HPAA was analyzed through whole-cell bioconversion experiments. The results were compared in Fig. 4. The in vivo efficiency of the evolved pathway was 3.28 ± 0.12 mg/L/h/OD, while the in vivo efficiency of the parent pathway was 1.91 ± 0.05 mg/L/h/OD, indicating a 72% increment in activity level. This result is consistent with the in vitro crude enzyme assays. This result indicates that the evolved pathway was more efficient at converting 4-hydroxyphenylpyruvate to 4HPAA than the wild-type pathway both in vitro and in vivo.

Directed evolution is the most commonly used approach used to significantly improve the performance of pathway enzymes. Applying direction evolution, we isolated mutant variants with improved activity than the wild-type enzymes. The best combination of the ARO10 and FeaB mutants resulted in a 90% increase in the production of 4HPAA. The increased 4HPAA titer may be as a result of increases in the activity of pathway enzymes.

**Fig. 5:**
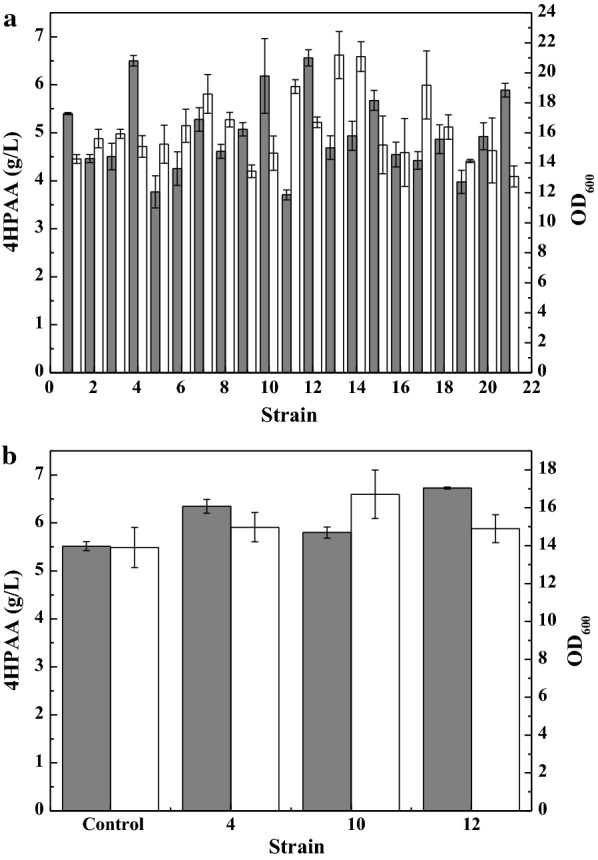
Growth (white bar) and 4HPAA production (gray bar) for *E. coli* DOPA-30N harboring the TIGR-mediated gene cluster of the evolved S. cerevisiae ARO10* and E. coli feaB*. **a** Colonies with lighter colors from the TIGR library; **b** strains with higher levels of 4HPAA. Cells were grown at 30 °C and 200 rpm for 72 h. See “Methods” for other details. *E. coli* DOPA-30N (pBbB2k-ARO10*-L-feaB*) was set the control. The data represent the means of three replicates, and error bars represent standard deviations

The unbalanced expression of multiple genes may result in the accumulation of toxic metabolic intermediates, thereby reducing product titers. Thus, Pfleger et al. [23] developed a combinatorial engineering approach for coordinating the expression of cascade enzymes by generating libraries of TIGRs. They applied the TIGR approach to balance gene expression in the MEV pathway, resulting in a sevenfold increase in the production of mevalonate. We also applied the TIGR approach to improve the production of zeaxanthin [19] and pinene [29]. Thus, we constructed a library of TIGRs to balance the expression of the evolved ARO10* and feaB*. The library of TIGRs was inserted between the two genes. A total of 1080 colonies were used for the assay of 4HPAA production in deep-well microplate cultures. Twenty colonies with lighter colors were selected for further shake flask analysis. As shown in Fig. 5a, strains nos. 4, 10, and 12 produced higher levels of 4HPAA. We again assayed the production of 4HPAA in the three strains (Fig. 5b). Strain no. 12 produced higher levels of 4HPAA, 6.58 g/L (Fig. 5b). Regulation of TIGR resulted in a 16.7% increase in the production of 4HPAA compared with the fusion evolved pathway. The TIGR in strain no. 12 was sequenced and the results are presented in Additional file 1: Table S2.

### Dynamic regulation of the biosynthetic pathway

In the above study, the inducible expression system was used to regulate the biosynthetic pathway of 4HPAA. However, this system has some disadvantages, including leaky expression, lack of dynamic control, and the prohibitively high cost of inducers that are associated with large-scale production. QS functions to control gene expression in response to cell density, and has been applied to several biosynthetic pathways to produce glucaric acid, myo-inositol, shikimate [15], β-lactamase exoenzyme [30], isopropanol [31], and bisabolene [32] in *E. coli*, and para-hydroxybenzoic acid in *Saccharomyces cerevisiae* [33]. The Lux and Esa QS systems are the main QS systems that have been reported to date. The QS system requires two regulatory elements, a signaling molecule (also known as an autoinducer) that is constitutively produced, and a receptor protein that binds the autoinducer and acts as a transcriptional activator or repressor. The Esa QS system from *P. stewartii*, has been engineered for both transcriptional activation and repression by regulating EsaR binding sites on the promoter [34]. In the absence of signal molecules, 3-oxohexanoyl-homoserine lactone (AHL), the transcriptional regulator in EsaRI70V, binds the P_esaS_ or P_esaR_ promoter and activates or represses transcription, respectively [15, 34, 35]. Thus, we constructed an Esa QS plasmid, pZBK-P_esaS_IC-P_esaR_AS, with both activating and repressing functions (Additional file 1: Figure S1A). To characterize the function of the QS circuit, GFP and mCherry genes were successively inserted into the multiple cloning site 1 (MSC1) and MSC2 to obtain pZBK-P_esaS_-GFP-P_esaR_-mCherry (Additional file 1: Figure S1B). The relative fluorescence of GFP decreased with cell growth (Additional file 1: Figure S1C). From Additional file 1: Figure S1C, we can also see that the relative fluorescence of mCherry increased with cell growth. These results demonstrate that the P_esaS_-controlled gene was indeed repressed and that the P_esaR_-controlled gene was activated by the induction of AHL in response to cell density.

**Fig. 6:**
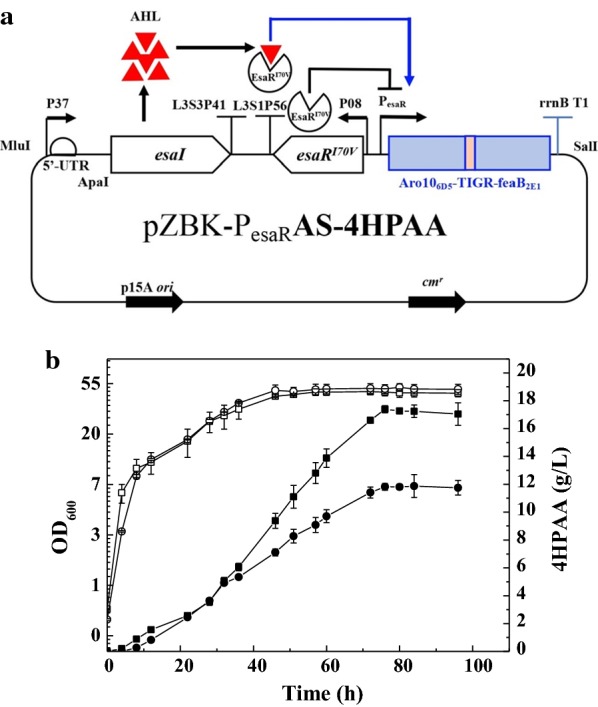
**a** The quorum-sensing (QS) system for 4HPAA production. In the absence of 3-oxohexanoyl-homoserine lactone (AHL, red triangle), the transcriptional regulator EsaR^I70V^ binds the P_esaR_ promoter and represses the transcription of the pathway enzymes. *Esa*I proteins synthesizes autoinducer AHL, which can diffuse in or out cells and binds to the transcriptional regulator EsaR^I70V^. At the threshold level of AHL, which accumulates in proportion to cell density, the transcriptional regulator EsaR^I70V^ binds to AHL, leading to dissociation of EsaR^I70V^from the P_esaR_ promoter, and the EsaR^I70V^-AHL complex activates the transcription of the pathway enzymes. **b** Fed-batch cultures of *E. coli* DOPA-30N harboring the QS-controlled (squares) and the statically controlled (circles) pathway for 4HPAA production. Growth (open: open square, open circle); 4HPAA concentration (black: filled square, filled circle). Experiments were conducted in duplicate, and measurements are presented as the means with standard deviations

Thus, the TIGR-mediated gene cluster of the evolved *ARO10*_*6D5*_ and *feaB*_*2E1*_ was cloned into the MCS2 site of the Esa QS plasmid pZBK-P_esaS_IC-P_esaR_AS to obtain pZBK–P_esaR_AS-4HPAA, which was then transferred into *E. coli* DOPA-30N. In this strain, the pathway was controlled by the P_esaR_ promoter, indicating that the expression of the pathway was activated by the induction of AHL in response to cell density (Fig. 6a). To compare the production of 4HPAA between the recombinant strain harboring the QS-controlled and the statically controlled pathway, fed-batch cultures were conducted in 2-L bioreactors. The results are presented in Fig. 6b. The engineered strain harboring the statically controlled pathway produced 4HPAA of 11.88 ± 0.82 g/L with a molar yield of 14.1% at 84 h. The engineered strain harboring the QS-controlled pathway produced higher level of 4HPAA titer, which achieved 17.39 ± 0.26 g/L with a molar yield of 23.2% (mol/mol) at 76 h. This indicates that QS-regulation increased the production of 4HPAA by 46.4% compared to the statically controlled pathway.

A Lux QS system from *Vibrio fischeri* has been used to construct a toggle switch for redirecting metabolic flux from the TCA cycle to the production of isopropanol, resulting in approximately a threefold increase in the titer [31]. This Lux QS system still requires external IPTG inducer to turn on LuxR/LuxI expression. Recently, an inducer-free Lux QS system has been used to dynamically regulate heterologous mevalonate and bisabolene biosynthetic pathways to improve the inducer-free production of bisabolene by 44% in *E. coli* [32]. An EsaR-P_esaS_ QS system from *P. stewartii* has been constructed to improve glucaric acid in *E. coli* by dynamically repressing glycolysis, leading to a fivefold increase in its titer [15]. In their study, the QS system was used to repress the competing pathway, driving the flux toward the biosynthetic pathway. In our study, we constructed an Esa-P_esaR_ activation QS system to dynamically control the biosynthetic pathway for the inducer-free production of 4HPAA, leading to a 46.4% improvement in the titer. This is the first report on an Esa activation QS system for dynamically regulating a biosynthetic pathway. The 4HPAA titer obtained in this study is 18.7-fold the maximum reported in literatures (0.93 g/L).

## Conclusion

We designed three biosynthetic pathways of 4HPAA based on tyrosol synthesis. Of these pathways, the yeast Ehrlich pathway exhibited the highest efficiency for the production of 4HPAA. To increase the pathway efficiency, the yeast Ehrlich pathway enzymes were directedly evolved using error-prone PCR. After directed evolution, two ARO10 and FeaB variants that outperformed the wild-type enzymes were obtained. These mutations increased the efficiency for converting 4-hydroxyphenylpyruvate to 4HPAA both in vitro and in vivo. The TIGR regulation for the evolved pathway enzymes further improved 4HPAA production. An Esa QS circuit with both activating and repressing functions was developed. The Esa-P_esaR_ activation QS system was used to dynamically control the biosynthetic pathway of 4HPAA, which yielded 17.39 ± 0.26 g/L 4HPAA without addition of external inducers. The titer is the highest value that has been obtained to date.

## Additional file

**Additional file 1: Figure S1.** (A) Esa quorum-sensing (QS) plasmid. (B) Activation and repression of genes using quorum-sensing circuit. **Table S1.** Mutations on the evolved gene. **Table S2.** The sequence of the TIGR.
