# Supplementary material for: Combining directed evolution of pathway enzymes and dynamic pathway regulation using a quorum-sensing circuit to improve the production of 4-hydroxyphenylacetic acid in Escherichia coli
Source: Biotechnol Biofuels. 2019 Apr 23;12:94. doi: 10.1186/s13068-019-1438-3 (PMC6477704; doi:10.1186/s13068-019-1438-3)
Supplement: Supplementary file 1 — Additional file 1: Figure S1. (A) Esa quorum-sensing (QS) plasmid. (B) Activation and repression of genes using quorum-sensing circuit. Table S1. Mutations on the evolved gene. Table S2. The sequence of the TIGR. [file 13068_2019_1438_MOESM1_ESM.docx]

**Combining directed evolution of pathway enzymes and dynamic pathway regulation using a quorum-sensing circuit to improve the production of 4-hydroxyphenylacetic acid in *Escherichia coli***

*Yu-Ping Shen, Lai San Fong, Zhi-Bo Yan, Jian-Zhong Liu**

*Institute of Synthetic Biology, Biomedical Center,* *Guangdong Province Key Laboratory of* *Improved Variety Reproduction in Aquatic Economic Animals and South China Sea Bio-Resource Exploitation and Utilization Collaborative Innovation Center, School of Life Sciences,* *Sun Yat-sen University, Guangzhou 510275, China*

*Corresponding author: Institute of Synthetic Biology, School of Life Science, Sun Yat-Sen University, Guangzhou 510275, P.R. China. Phone: +86-20-84110115. Fax: +86-20-84036461. *E-mail address*: lssljz@mail.sysu.edu.cn (J. Z. Liu)


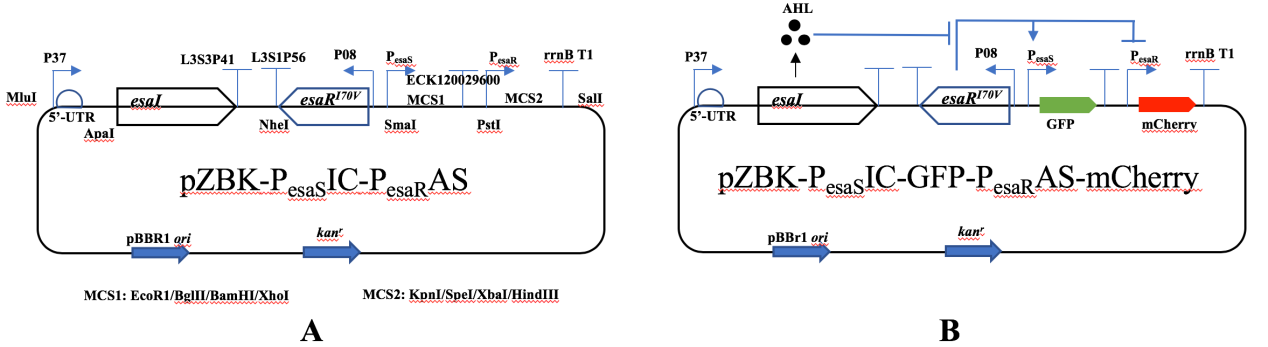

Figure S1. (A) Esa quorum-sensing (QS) plasmid. (B) Activation and repression of genes using quorum-sensing circuit. The QS signaling molecule (3-oxohexanoyl-homoserine lactone, AHL) is produced by the AHL synthase encoded by *esaI*. In the absence of AHL or at low AHL concentration, the transcriptional regulator EsaR^I70V^ binds the P_esaS_/or P_esaR_ promoter and activates the transcription of GFP, and/or represses the transcription of mCherry. As cell density increases, AHLs accumulate, resulting in disruption of EsaR^I70V^ binding and repressing of the transcription of GFP, and/or activating of the transcription of mCherry. (C) Gene expression from P_esaS_/or P_esaR_ promoter in the Esa quorum-sensing circuit. As cell growth, the transcription level of GFP driven by the P_esaS_ promoter decreased, and the transcription level of mCherry driven by the P_esaR_ promoter increased.

Table S1. Mutations on the evolved gene

| Plasmid | gene | Base mutation | Amino acid mutaion |
| --- | --- | --- | --- |
| 2E1 | *ARO10* | C1869T | No |
|  | *feaB* | T72G, T1195C, A1491T | I24M |
| 4F3 | *ARO10* | A1682G, A1842G | No |
|  | *feaB* | C165G, A609T, T1296 | N55K |
| 6D5 | *ARO10* | T412C, A653G, T948C, A1815T | F138L, D218G |
| 9F5 | *ARO10* | T695C, A1599G, A1776G | No |
| 10A3 | *ARO10* | T989C, G1351A, A1638G | V451I |
| 10G5 | *ARO10* | C1722T | No |

Table S2. The sequence of the TIGR

GCCTAGCAAGATCTCCTGATCCCGGTGCGCGACCACCCGGACATCTGCATAGTCTGGGCCAGTCTGAGGACTGGCGGATCAGGGCCTTGAATTTACAGTATTTTAGTGGCCTTACGCTATACTATTCGGTCACCTTATCCGCTCAAGACATGCACTCGGAACGCATCTAGGGTACCGCAGATACTGTATCC
